# Bado type III Monteggia fractures have a high injury- and treatment-related complication rate: a single center study of 73 fractures

**DOI:** 10.2340/17453674.2024.42111

**Published:** 2024-10-14

**Authors:** Kaj ZILLIACUS, Yrjänä NIETOSVAARA, Ilkka HELENIUS, Niko KÄMPPÄ, Ilkka VUORIMIES, Petra GRAHN

**Affiliations:** 1 Department of Pediatric Orthopedics and Traumatology, New Children’s Hospital, Helsinki University Hospital, University of Helsinki, Helsinki; 2 Department of Orthopedics and Traumatology, HUS Helsinki University Hospital, University of Helsinki, Helsinki; 3 Department of Hand Surgery, HUS Helsinki University Hospital, University of Helsinki, Helsinki; 4 Department of Radiology, HUS Diagnostic Center, University of Helsinki and Helsinki University Hospital, Helsinki; 5 Department of Pediatric Surgery, Kuopio University Hospital, University of Eastern Finland, Kuopio, Finland

## Abstract

**Background and purpose:**

Monteggia fractures can be problematic injuries. The aim of this population-based study is to evaluate the risk of complications according to the Bado types, clinical outcome, and incidence.

**Methods:**

72 children (median age 6, range 2–11 years) with 73 Monteggia fractures treated during 2014–2022 were identified from the institutional fracture register. Timing of diagnosis, complications, and method of treatment were registered. Outcomes were assessed at mean 4 years (1–9) follow-up in 68 (94%) children. The census population (< 16 years old) in Helsinki metropolitan area during the study period was assessed.

**Results:**

Bado types I (n = 43) and III (n = 27) comprised all but 3 of the fractures. Diagnosis was made on admission in 57, and with a 1–8-day delay in 16 children. 8 children had sustained an associated nerve injury. 35 children were treated operatively, 7 after failed closed treatment. 4 reoperations were performed, including 3 ulnar osteotomies. The risk of complications (odds ratio [OR] 4.9, 95% confidence interval [CI] 1.7–14) and closed treatment failures (OR 12.3, CI 1.3–118) was higher in Bado type III than in type I injuries. 60 children attended for clinical follow-up, all had congruent radio-humeral joints and full range of elbow and forearm motion. Mean PedsQL was 94 (72–100) and QuickDash 3 (0–13). 8 additional children reported normal elbow functions by phone. The calculated mean annual incidence of Monteggia injuries was 2.9/100,000 children.

**Conclusion:**

Monteggia fractures are rare (2.9/100,000 yearly). Bado type III injuries are associated with a high risk of complications.

Pediatric Monteggia fracture dislocations primarily affect 5–7-year-old children and constitute about 1% of all children’s fractures with a reported incidence of 0.4:100.000 [1-4]. Open fractures and associated nerve injuries are rare [5,6].

Management of Monteggia fracture dislocations includes closed or open reduction of the ulnar fracture with or without internal fixation, concomitant reduction of the radial head, immobilization in a well-contoured long arm cast and close follow-up to minimize the risk of overlooking loss of reduction [3,7,8]. Monteggia injuries are frequently overlooked (16–50%) in children, delaying treatment to the point where closed reduction of the radiohumeral joint and the ulnar fracture is no longer possible [2,9**-**12]. Most of these cases can be salvaged with an osteotomy of the malunited ulna and open reduction of the radiohumeral joint [2,13].

Studies on pediatric Monteggia fracture focus mostly on patients with delayed or missed treatment, reporting only on short-term (< 6 month) outcomes [3,4,7,14]. We aimed to evaluate the risk of complications and treatment outcomes in mid-term follow-up across different Bado types. Additionally, we sought to estimate the incidence of Monteggia fractures in the Helsinki Metropolitan area.

## Methods

### Design and data source

This single-center, registry-based clinical study was conducted at the HUS (Helsinki University Hospital) New Children’s Hospital. HUS New Children’s Hospital, a level 1 pediatric trauma center, serves a catchment area of 1.7 million inhabitants. The treatment of Monteggia fractures within this catchment area is centralized to this hospital. All surgeries are performed under the supervision of pediatric orthopedic or pediatric hand surgeons. All trauma radiographs in under-16-year-old children taken at the hospitals of our primary catchment area are reviewed weekly at our institution by a team of pediatric radiologists and pediatric orthopedic surgeons to verify or correct the initial diagnosis and treatment.

### Parameters

**Figure 1:**
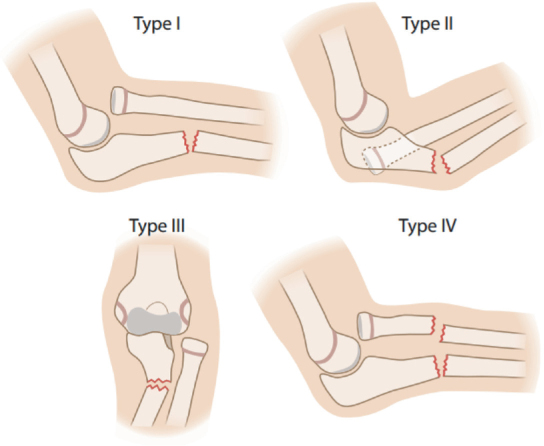
The 4 different types of Monteggia fracture dislocations as classified by Bado et al. [15]. Dislocation of radial head is anterior in Type I, posterior in Type II, and lateral in Type III. Type IV injuries include a fracture of radius.

Between 2014 and 2022, children with Monteggia fractures (ICD-10: S52.0–S52.7 + S53.0–S53.01) were identified from the institutional fracture register (Kids Fracture Tool, BCB Medical, Turku, Finland). STROBE guidelines were followed (https://www.strobe-statement.org/). Patient demographics including age, sex, injury type, and hand dominance were documented. A radiologist with experience in pediatric trauma re-evaluated all radiographs for Bado classification [15] (Figure 1) and fracture morphology. Possible delay of correct diagnosis was registered in days.

### Outcomes

At follow-up, active elbow extension and flexion, forearm pronation and supination, and elbow carrying angle were measured using a goniometer. Grip strength was recorded with a Jamar Hydraulic Hand Dynamometer (Lafayette Instrument Company, Lafayette, IN, USA). Elbow stability was assessed using the valgus stress test and chair test [16]. Standard elbow and forearm lateral and anteroposterior radiographs were taken and assessed for fracture healing, congruency of the radiohumeral joint, and also any associated pathology.

Clinical outcome was assessed using the functional evaluation system introduced by Bruce et al. [17] (Table 1). Patient-reported outcomes (PROMs) were assessed using the Pediatric Quality of Life Inventory (PedsQL) [18,19], the PesdQL Pediatric Pain Questionnaire (PedsQL Pain), and the Quick Disabilities of the Arm, Shoulder and Hand (QuickDASH) [20**-**22]. Cosmetic appearance was assessed using a visual analog scale (VAS) from 0–10 with 10 being the best possible outcome [18,19].

**Table 1:** Criteria for outcome in Monteggia fracture dislocations adapted from Bruce et al. [17]

Range of motion (60 points)
Number of points of ROM = 60 – (percentage impairment of upper extremity x 0.6)
Activities of daily living (ADL) and work status (20 points)
20 – Function equal to opposite arm
15 – Independent ADL; no more than 2 work handicaps
10 – Unable to do more than three ADL; 3 or more work handicaps; occupational change required
5 – Unable to do 4 or more ADL; occupational disability
Pain (15 points)
15 – No pain
13 – Annoying pain with no compromise of activity
10 – Pain interfering with activity
5 – Pain preventing some activity
0 – Pain causing outcries and preventing activities
Anatomy
1 – Acceptable cosmetic appearance
1 – No clinical angulation
1 – No Clinical displacement
1 – Clinical change of carrying angle less than 10 degrees
1 – Roentgenographic union
Results
Excellent: 96–100
Good: 91–95
Fair: 81–90
Poor: < 80

The under-16-year-old census population in Helsinki metropolitan area during the study period between 2014 and 2022 was 2,441,653 children as reported by Statistics Finland (stat.fi).

### Statistics

Statistical analyses were performed using R version 4.3.0 (R Foundation for Statistical Computing, Vienna, Austria). Continuous variables were assessed using Q–Q plots and the Shapiro–Wilk test. As these indicated non-normality, medians with interquartile ranges (IQR) and 95% confidence intervals (CI) are used to present data. Categorical variables are presented as (n). Groupwise comparisons were only performed between the Bado type I and III subgroups as the Bado type II and IV subgroups suffered from insufficient numbers of participants for meaningful statistical analyses. A median difference between subgroups was calculated and basic bootstrapping was employed to calculate CIs. Categorical variables were analyzed using Fisher’s exact test. In the groupwise analyses of complications, odds ratios (OR) with CIs were calculated. A P value < 0.05 was considered statistically significant. Estimation of incidences of Monteggia fractures in the pediatric population was calculated over the years 2014–2022.

### Ethics, data sharing plan, funding, and disclosures

Written informed consent was obtained from guardians and participants. The study protocol was approved by the HUS Regional Committee on Medical Research Ethics (approval number: 78/1801/2020). The authors report no conflict of interest. Complete disclosure of interest forms according to ICMJE are available on the article page, doi: 10.2340/17453674.2024.42111

## Results

### Patients, fracture demographics, and diagnoses

**Figure 2:**
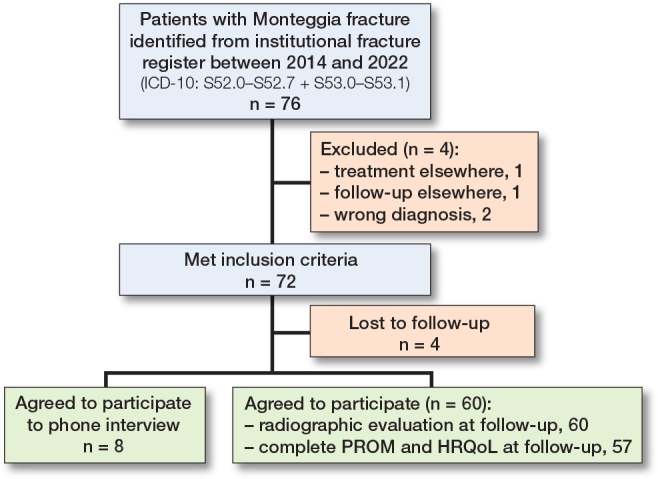
Flowchart of patients included and excluded in the study. PROM = patent-reported outcome measure. HRQoL = health-related quality of life.

76 children were identified. 4 patients were excluded due to initial treatment elsewhere or incorrect diagnosis. 72 patients with 73 Monteggia fractures were included in the study. 60 of the 72 (83%) patients agreed to attend a clinical and radiological follow-up. An additional 8 children (11%) were interviewed by phone. 4 children were lost to follow-up (Figure 2). At the time of injury, the mean age of the 72 included patients (73 fractures; 37 males; 68 right-sided) was 6.4 years (range 2.5–11.2 years). The most common mechanism of injury, occurring in 39 out of 73 cases, was a fall from over 1 m in height (Table 2). Most fractures were either Bado type I (43) or type III (27) injuries. 2 children had an open fracture, and an elbow dislocation was reduced in 3 children. There were 2 concomitant medial epicondyle fractures, 1 distal radius buckle fracture, and 1 non-displaced lateral humeral condyle fracture. 8 patients had sustained a fracture-related nerve injury: radial nerve (6), ulnar nerve (2), both ulnar and median nerve (2). 7 children’s nerve injuries were temporary, 1 child with a radial nerve injury was lost to follow-up before 1 year from injury. An 11-year-old boy re-fractured his ulna (Bado type II) 6 months after the primary injury. The diagnosis was initially missed from the primary radiographs in 16/73 (22%) injuries, causing a median treatment delay of 6 days (range 1–8 days). The delayed diagnosis did not influence the treatment method (P = 0.8) or worsen outcomes (QDASH, P = 0.7, PedsQl physical functioning, P = 0.3) compared with all fractures. However, injuries with delayed primary diagnosis were more likely to experience failure of closed treatment (OR 8.1, CI 1.3–48.6, P = 0.03).

**Table 2:** Demographic and clinical characteristics of 73 Monteggia fracture dislocations in 72 children sorted by Bado type

Factor	Bado I (n = 43)	Bado II (n = 2)	Bado III (n = 27)	Bado IV (n = 1)	Total (n = 73)
Sex					
Male	19	2	15	1	37
Female	24	0	12	0	36
Age at injury, years					
median	6.2	8.1	5.7	10.6	6.1
IQR	5.1–7.7	6.5–9.7	4.8–7.1	–	5.0–7.7
Mechanism of injury					
Fall from height	24	0	14	1	39 (53)
Ground-level fall	13	0	10	0	23 (32)
Sports-related	6	2	3	0	11 (15)
Diagnosis (ICD-10)					
S52.2	26	0	7	1	34 (47)
S52.0	15	2	19	0	36 (49)
S52.0 and S52.2	2	0	1	0	3 (4.1)
Fracture type					
Incomplete	24	0	3	1	28 (38)
Transverse	3	1	5	0	9 (12)
Oblique	14	0	4	0	18 (25)
Comminuted	2	1	15	0	18 (25)

IQR = interquartile range. S52.0 = proximal ulna fracture. S52.2 = fracture of the ulnar shaft.

### Treatment

**Figure 3:**
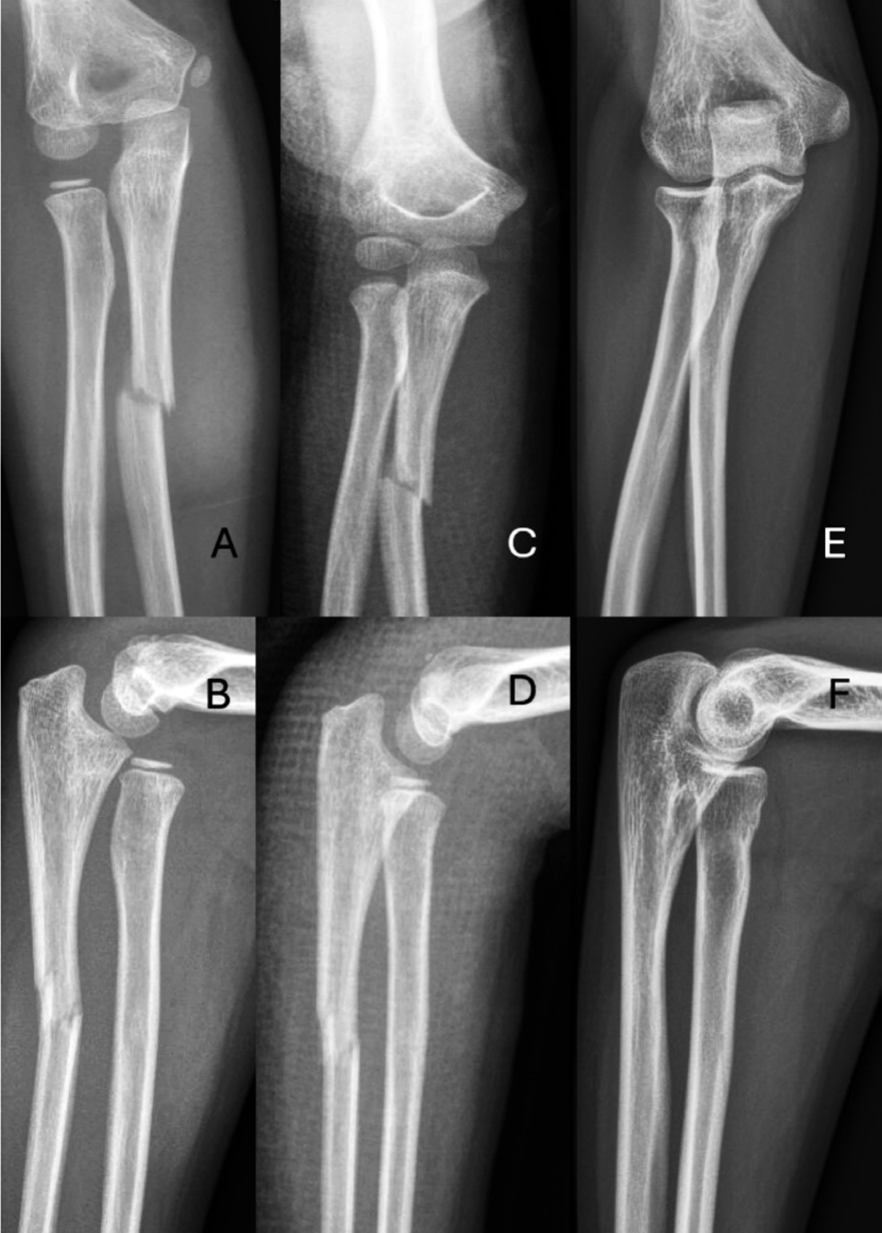
A 7-year old girl with Monteggia fracture Bado type I (A, B) treated with closed reduction and cast (C, D). Injury had healed fully radiographically and functionally 8 years after injury (E, F).

**Figure 4:**
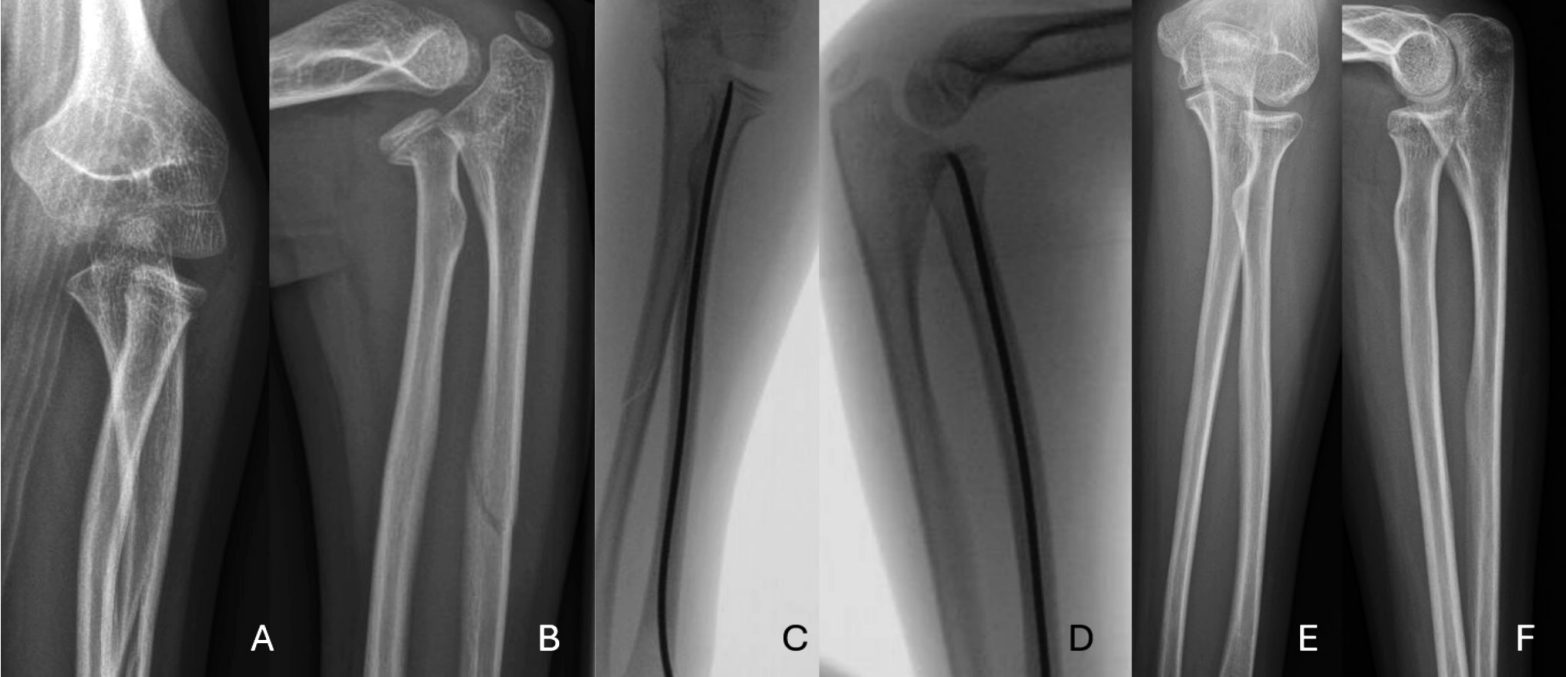
A 10-year-old boy, with left hand injury. In the radiographs a proximal fracture of the radius and anterior luxation of the radiohumeral joint and ulnar diaphyseal fracture were registered (A, B). This was classified as Bado type IV Monteggia fracture. Injury was treated with flexible intramedullary nail (C, D). 5 years later after removal of the nail, the injury had recovered fully radiologically and clinically (E, F).

45 (62%) injuries were primarily treated with closed reduction and a long arm cast. According to Bado types, 65% of type I (Figure 3, see Appendix), 50% of type II, 59% of type III, and none of the type IV fractures (Figure 4, see Appendix) were primarily treated with closed reduction and cast. Among the 28 (38%) surgically treated patients, primary open reduction of the radiohumeral joint was performed in 6 children. Annular ligament reconstruction was not performed in any patients. 1 patient developed a postoperative superficial surgical site infection, which healed with peroral antibiotics.

### Complications

Primary treatment failure was observed in 11 cases (15%) after a median follow-up of 8 days (range 1–54 days) in 7/38 nonoperatively and 4/35 operatively treated patients. Bado type III injuries were at the highest risk of closed treatment failure (OR 12.3, CI 1.3–118) and complications (OR 4.9, CI 1.7–14) (Table 3). All 8 treatment failures in Bado III injuries involved a proximal comminuted (7 patients) or a proximal oblique ulnar fracture (1 patient) morphology.

**Table 3:** Number of complications by Bado group: comparison between groups Bado I and Bado III

Factor	Bado I n = 43	Bado II n = 2	Bado III n = 27	Bado IV n = 1	Bado III vs Bado I OR (CI)
Missed primary diagnosis	9	1	6	0	1.1 (0.3–3.5)
Transient nerve injury	3	0	4	1	2.3 (0.5–11.3)
Refracture	0	1	0	0	–
Successful conservative					
treatment	27	0	11	0	0.4 (0.2–1.1)
Failed	1	1	5	0	12.3 (1.3–118)
Operative treatment	16	2	16	1	2.5 (0.9–6.6)
Failed	1	0	3	0	3.2 (0.3–35.1)
Superficial wound infection	0	0	1	0	–
All complications	14	3	19	1	4.9 (1.7–14.0)

OR (odds ratio) were not calculated for Bado II and IV due to small sample size.

**Figure 5:**
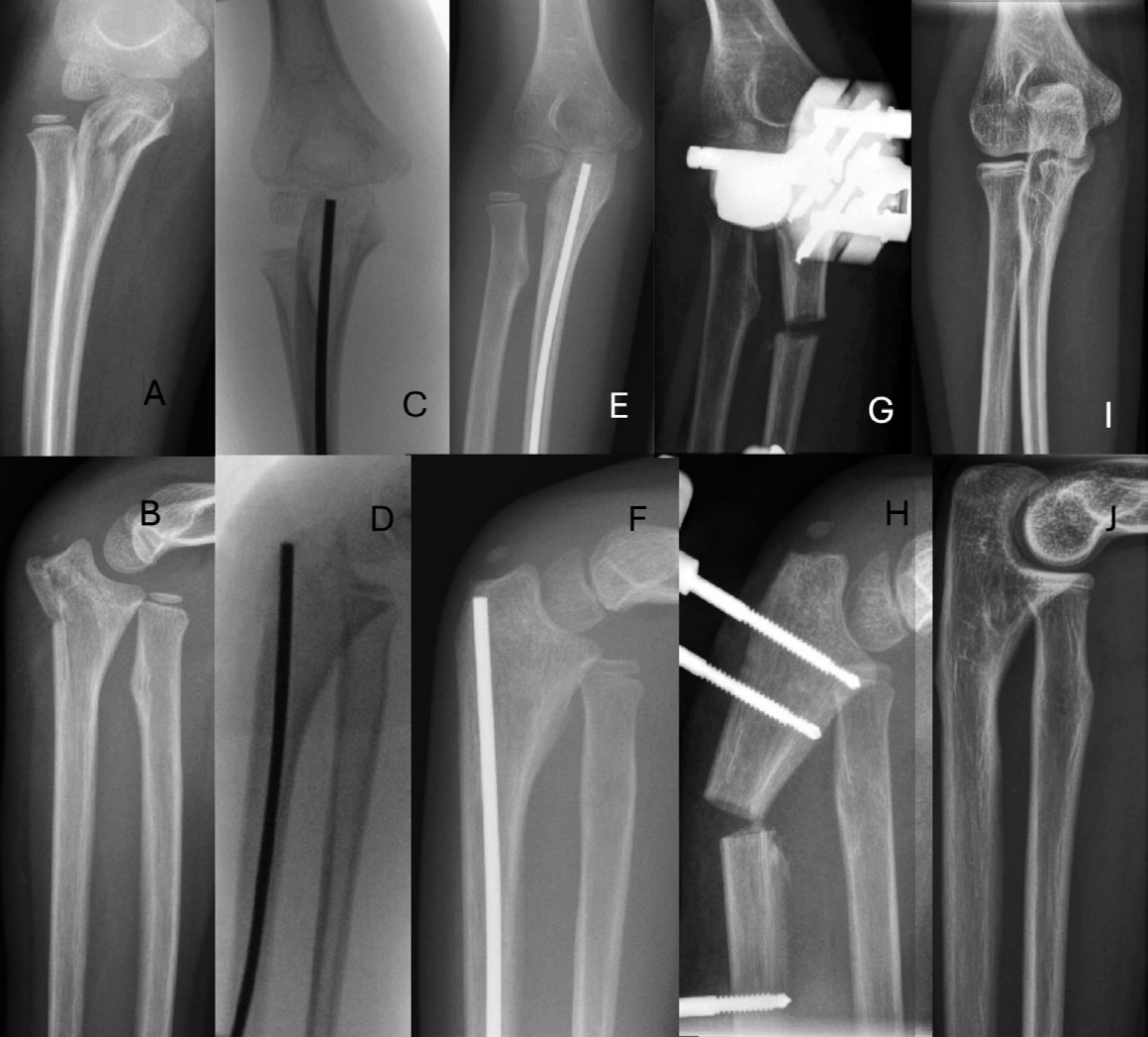
A 5-year-old girl with a Bado type III Monteggia fracture (A, B) treated with closed reduction of the radiohumeral joint (RHJ) and flexible intramedullary nailing of the proximal ulna fracture (C, D). 3 months from surgery persistent lateral dislocation of the RHJ was observed (E, F) and an ulnar osteotomy with an open reduction of the RHJ was performed as described by Hasler et al. [13] (G, H). Radiographs 6 years later show a congruent RHJ and normal alignment of the ulna (I, J).

Treatment failures for the operatively treated patients included unsatisfactory reduction at the initial procedure in 3 patients (1 Bado I and 2 Bado III) and loss of reduction in 1 patient (Bado III). All 11 patients who experienced primary treatment failure underwent surgical intervention, with 3 requiring ulnar osteotomy and open reduction of the radiohumeral joint according to the technique described by Hasler et al., performed 7–17 weeks after the injury (Figure 5) [13].

### Outcomes

Complete fracture consolidation and congruent radiohumeral joints were observed in all 60 patients who attended the radiographic and clinical follow-up at mean 4 years (range 1–9 years). The patients presented with full active range of motion in the elbow (median 160°, CI 155–160, IQR 155–160) and forearm (median 165°, CI 165–170, IQR 160–170), symmetrical carrying angles (median 10°, CI 10–12.5, IQR 5–15), and comparable grip strength (median 15 kg, CI 12–16, IQR 10–19) between the injured and uninjured sides (Table 4). The valgus stress test was positive in 3 asymptomatic patients treated nonoperatively. All 60 patients had an excellent (58) or a good (2) result according to the Bruce et al. criteria [18].

**Table 4:** Comparison between outcomes in Bado type I and III fractures

Factor	Bado I median (IQR)	Bado III median (IQR)	Difference in medians (CI)
QuickDASH	0 (0 to 0)	0 (0 to 0)	0 (0 to 2.1)
Current pain	0 (0 to 0)	0 (0 to 0)	0 (0 to 0)
Worst pain	0 (0 to 0)	0.5 (0 to 0)	0 (0 to 1)
PedsQL score			
Total score	90.2 (79.9 to 95.1)	84.2 (79.6 to 92.4)	4.5 (–1.9 to 11.2)
Physical Functioning	96.9 (90.6 to 100)	92.2 (90.6 to 99.2)	3.1 (–3.2 to 9.3)
Cosmetic VAS	10 (10 to 10)	10 (8.5 to 10)	0 (0 to 0)
Difference between affected and healthy side in			
grip strength, kg	0 (–2 to 1)	0 (–1 to 2)	0 (–1 to 2)
extension–flexion AROM,°	0 (0 to 5)	0 (–5 to 0)	0 (–5 to 0)
carrying angle, °	0 (0 to 0)	0 (–5 to 0)	0 (–2.5 to 0)
pro-supination AROM, °	0 (–5 to 5)	0 (–5 to 0)	0 (–5 to 0)

CI = confidence interval, IQR = interquartile

PROM scores were all within normal range with a median QuickDash 0 (CI 0, IQR 0–1.8), median PedsQL 87 (CI 82.6–92.4, IQR 79.3–93.5), and a median PedsQL sub-score of physical functioning 96.7 (CI 93.4–100, IQR 90.6–100). The median PedsQL of present and worst pain was 0 (CI 0, IQR 0–0 and 0–1 respectively) (Table 4). All 68 patients were satisfied with the appearance of the arm with a median cosmetic VAS score of 10 (CI 10, IQR 10–10).

### Incidence

The mean incidence of Monteggia fracture dislocation over the study period in HUS New Children’s hospitals catchment area was 2.9/100,000 (annual variation 1.5–5.7/100,000).

## Discussion

We investigated clinical, radiographic, and patient-reported outcomes in 94% of the children treated for Monteggia fracture dislocations in Southern Finland between 2014 and 2022. Our study identified a relatively high risk (22%) of primarily missed Monteggia fracture dislocations, which did not lead to clinically significant consequences. We found Bado type III injuries to have the highest risk of closed treatment failure (31%).

Monteggia fracture dislocations are initially missed in 16–50% of cases [2,9,12]. A missed diagnosis is typically defined as a delay in correct diagnosis of more than 4 weeks [2,9]. In our series, we identified a diagnostic delay in one-fourth of the cases within our catchment area. Due to our weekly quality control meetings, none of our cases experienced a diagnostic delay of more than 8 days. Hubbard et al. reviewed missed Monteggia fractures and reported that a delay of only 2 weeks can present a serious clinical challenge, often necessitating operative intervention. The longer the delay, the less likely it is to achieve and maintain a congruent radiohumeral joint. Consequently, the most common long-term complications are sequelae following incongruence of the radiohumeral joint [2]. Although the failure rate after closed reduction and cast treatment was higher in patients with a slight diagnostic delay in our study cohort, it did not impair the final outcome, which was good to excellent in all patients. We believe this finding underscores the importance of quality control meetings governed by pediatric trauma centers.

**Figure 6:**
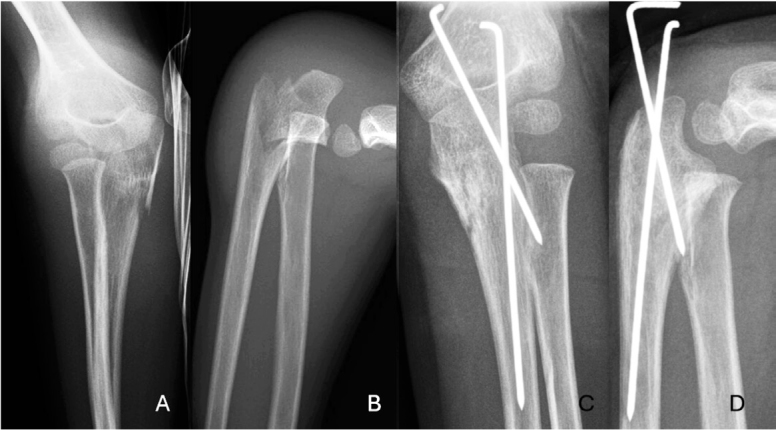
A 5-year-old boy, with left hand injury. In the radiographs a proximal ulnar fracture and posterior luxation of the radiohumeral joint was registered (A, B). This was classified as Bado type II Monteggia fracture. Injury was treated with Kirschner wires, which were afterwards removed (C, D). Injury had healed fully 7 years later when the patient was interviewed via phone.

According to previous studies Bado types I and III constituted 88–91% [3,4,23] of all pediatric Monteggia injuries. Our results are in line with these earlier reports, although we found type II (2.7%)(Figure 6, see Appendix) and IV (1.4%) injuries to be even more rare than earlier reported. The majority of pediatric Monteggia fracture dislocations can be treated by closed means, with similar outcomes reported between closed and operative treatment groups in short-term studies [3,7].The key to a successful outcome is the reduction of the radiohumeral joint [9,11]. Hart et al. reported a 14% risk of loss of reduction in non-surgically treated patients and found that surgical treatment was more often required for Bado type III and IV injuries [7]. Our study showed a similar rate (15%) of loss of reduction following closed treatment and similarly identified Bado type III and IV injuries as often requiring surgical intervention. Bado type III injuries appear more prone to complications, leading Nuiding et al. to recommend primary internal fixation combined with reduction of the radial head for this injury type [24]. Consistent with our findings, they reported a high (22%) loss of reduction rate in Bado type III injuries following closed treatment. In contrast to the studies by Ramski et al. and Hart et al., who did not report any need for reoperations in altogether 185 primarily operatively treated patients, we found an 11% risk of primary treatment failure following operative treatment [4,7]. The reason for the difference could be in the patient cohort as nearly half of our patients required surgical treatment primarily, which is in great contrast to Foran et al. who managed 85% with closed means [3]. One of the reasons for the difference in treatment regimens might be in trauma mechanism with the majority of our patients experiencing a higher trauma energy (fall from heights). In the current study all 8 failed Bado III injuries included either a proximal comminuted or oblique ulnar fracture morphology. As a conclusion of our study and the aforementioned, internal fixation of ulnar fractures should be considered in Bado type III injuries.

Both the outcome of treatment and the incidence of Monteggia injuries in children is poorly documented, as most studies constitute small to medium sized retrospective case series with short follow-up times. Leonidou et al. reported excellent outcome in 32/40 patients in a mean follow-up of 4.6 years using the scoring system of Bruce et al. [17,23]. Subjective results have been assessed by Roper et al., who reported a mean DASH score of 5.9 in closed fractures and 13.1 in open fractures 4.6–15.6 months after treatment [14]. In this study, with a follow-up rate of 83%, nearly all patients (58/60) had an excellent outcome according to the scoring system by Bruce et al. [17]. In addition, all PROMs were within normal range regardless of the failed primary treatment in 11 of our patients. The only previous report on fracture incidence is by Landin [1], who reported an incidence of 0.4/100,000 for pediatric Monteggia fracture dislocations in a population-based study from Malmö Sweden during 1950–1979. He had only 2 patients with a Monteggia injury in his study. Our calculated incidence of Monteggia fracture dislocations was a mean 2.9/100,000 in a census population of nearly 2.5 million, which is significantly higher than that reported by Landin.

### Strengths and limitations

*Strengths.* We used prospectively collected population-based data from our institutional fracture register, which allowed us to identify all children with Monteggia injuries in Southern Finland over the study period and calculate the incidence of the injury. Additionally, we achieved a high follow-up rate and assessed mid-term outcomes clinically, radiographically, and with several different patient-reported outcome measures (PROMs). The calculated incidence can be regarded to be of high accuracy, as the vast majority of pediatric Monteggia injuries are treated at our institution because only a few private clinics offer 24/7 treatment of pediatric elbow trauma.

*Limitation.* There were only a small number of patients with Bado type II and IV injuries. As Monteggia fracture is a very uncommon injury, we have no treatment algorithm for this entity in our institution. Consequently, treatment decisions were based on the preferences of individual consultant pediatric orthopedic or hand surgeons, which can be seen as a limitation.

### Conclusion

Monteggia injuries are rare (2.9/100 000 annual incidence). Bado type III injuries carry a higher risk of fracture-related and treatment-related complications.

## Notes

KZ, IH, and PG planned the study. KZ gathered the data, analyzed it and wrote the manucript. IH, IV, NK, PG, and YN reviewed and modified the manuscpript. NK performed the statistical analysis. IV analyzed the radiographs.

Handling co-editors: Ivan Hvid and Robin Christensen

*Acta* thanks Niels Wisbech Pedersen and Marc Gerard Romijn for help with peer review of this manuscript
